# Poster Session II - A191 REAL-WORLD OUTCOMES OF DUPILUMAB FOR TREATMENT OF EOSINOPHILIC ESOPHAGITIS

**DOI:** 10.1093/jcag/gwaf042.191

**Published:** 2026-02-13

**Authors:** T T Hoang, A Pedicelli, V Avinashi, S Moosavi, G Ou, N Tardio, W Afif, H Ko

**Affiliations:** Medicine, The University of British Columbia, Vancouver, BC, Canada; McGill University, Montreal, QC, Canada; Medicine, The University of British Columbia, Vancouver, BC, Canada; Medicine, The University of British Columbia, Vancouver, BC, Canada; Medicine, The University of British Columbia, Vancouver, BC, Canada; McGill University, Montreal, QC, Canada; McGill University, Montreal, QC, Canada; Medicine, The University of British Columbia, Vancouver, BC, Canada

## Abstract

**Background:**

Eosinophilic esophagitis (EoE) is a chronic, immune-mediated inflammatory disease characterized by eosinophil-predominant inflammation causing esophageal dysfunction. Dupilumab, an anti–IL-4/13 monoclonal antibody, is the first biologic approved for EoE. Since Health Canada approval in 2023, real-world data remain limited.

**Aims:**

To evaluate clinical, endoscopic, and histologic outcomes in EoE patients treated with dupilumab.

**Methods:**

A multicenter retrospective review was conducted at four tertiary hospitals in Montreal and Vancouver. Included are biopsy-proven EoE adult and pediatric patients with ≥1 follow-up endoscopy ≥12 weeks after starting dupilumab. Primary outcomes are changes in Eosinophilic Esophagitis Endoscopic Reference Score (EREFS), Index of Severity for Eosinophilic Esophagitis (I-SEE), and peak eosinophil count per high-power field (eos/HPF) on pathology. Secondary outcomes include symptom improvement and post-treatment dilation.

**Results:**

Of the 49 patients on dupilumab, 36 met inclusion criteria (5 pediatric, 31 adult). Mean pre-treatment EREFS was 2.9±1.9, with a mean 70.1±31.4 eos/HPF on histology. After a mean treatment duration of 44.7±25.7 weeks, EREFS and eos/HPF improved to 1.1±1.1 and 4.5±9.0, respectively. We observed improvements in EREFS, I-SEE score, and eos/HPF post-dupilumab treatment (p < 0.001, Fig. 1). Subjective clinical improvement occurred in 97.2% of patients. During treatment, 37.1% and 90.1% discontinued PPI and inhaled steroids, respectively. Three patients stopped dupilumab for lack of efficacy, pregnancy, and initiation of a new biologic for vasculitis. Four patients had mild injection site reactions, and only three required post-treatment esophageal dilations.

**Conclusions:**

Our real-world data show dupilumab is a safe, highly effective therapy for EoE across pediatric and adult populations, improving endoscopic, clinical, and histologic disease activity.

**Funding Agencies:**

None

**A191 Table 1:** Baseline demographics (n = 36)

Age	20.8 ± 11.56 years	
Duration of symptoms before diagnosis	68.2 ± 96.9 months	
	n	%
Male sex	25	69.4
Esophageal dysphagia	33	91.7
Food bolus impaction	19	52.8
Chest pain	10	27.8
Heart burn	13	36.1
Regurgitation	10	27.8
Abdominal pain	9	25.0
Previous food bolus impaction requiring endoscopic intervention	8	22.2
Allergic rhinitis	23	63.9
Eczema	14	38.9
Asthma	16	44.4
Food allergies	20	55.6
Exclusion diet	19	52.8
Proton pump inhibitor	35	97.2
Topical steroid	33	91.7
Esophageal dilation	8	22.2

